# Characteristics and predictors of hospitalization and death in the first 11 122 cases with a positive RT-PCR test for SARS-CoV-2 in Denmark: a nationwide cohort

**DOI:** 10.1093/ije/dyaa140

**Published:** 2020-09-05

**Authors:** Mette Reilev, Kasper Bruun Kristensen, Anton Pottegård, Lars Christian Lund, Jesper Hallas, Martin Thomsen Ernst, Christian Fynbo Christiansen, Henrik Toft Sørensen, Nanna Borup Johansen, Nikolai Constantin Brun, Marianne Voldstedlund, Henrik Støvring, Marianne Kragh Thomsen, Steffen Christensen, Sophie Gubbels, Tyra Grove Krause, Kåre Mølbak, Reimar Wernich Thomsen

**Affiliations:** 1 Clinical Pharmacology and Pharmacy, Department of Public Health, University of Southern Denmark, Odense, Denmark; 2 Hospital Pharmacy Funen, Odense University Hospital, Odense, Denmark; 3 Department of Clinical Biochemistry and Clinical Pharmacology, Odense University Hospital, Odense, Denmark; 4 Department of Clinical Epidemiology, Aarhus University Hospital, Aarhus, Denmark; 5 Center for Population Health and Sciences, Stanford University, Stanford, CA, USA; 6 Department of Medical Evaluation and Biostatistics, Danish Medicines Agency, Copenhagen, Denmark; 7 Statens Serum Institut, Copenhagen, Denmark; 8 Department of Public Health—Biostatistics, Aarhus University, Aarhus, Denmark; 9 Department of Clinical Microbiology, Aarhus University Hospital, Aarhus, Denmark; 10 Department of Anaesthesia and Intensive Care Medicine, Aarhus University Hospital, Aarhus, Denmark

**Keywords:** COVID-19, SARS-CoV-2, infectious disease, epidemiology, population-based, predictors, hospitalization, death

## Abstract

**Background:**

Population-level knowledge on individuals at high risk of severe and fatal coronavirus disease 2019 (COVID-19) is urgently needed to inform targeted protection strategies in the general population.

**Methods:**

We examined characteristics and predictors of hospitalization and death in a nationwide cohort of all Danish individuals tested for severe acute respiratory syndrome coronavirus 2 (SARS-CoV-2) from 27 February 2020 until 19 May 2020.

**Results:**

We identified 11 122 SARS-CoV-2 polymerase chain reaction-positive cases of whom 80% were community-managed and 20% were hospitalized. Thirty-day all-cause mortality was 5.2%. Age was strongly associated with fatal disease {odds ratio [OR] 15 [95% confidence interval (CI): 9–26] for 70–79 years, increasing to OR 90 (95% CI: 50–162) for ≥90 years, when compared with cases aged 50–59 years and adjusted for sex and number of co-morbidities}. Similarly, the number of co-morbidities was associated with fatal disease [OR 5.2 (95% CI: 3.4–8.0), for cases with at least four co-morbidities vs no co-morbidities] and 79% of fatal cases had at least two co-morbidities. Most major chronic diseases were associated with hospitalization, with ORs ranging from 1.3–1.4 (e.g. stroke, ischaemic heart disease) to 2.6–3.4 (e.g. heart failure, hospital-diagnosed kidney disease, organ transplantation) and with mortality with ORs ranging from 1.1–1.3 (e.g. ischaemic heart disease, hypertension) to 2.5–3.2 (e.g. major psychiatric disorder, organ transplantation). In the absence of co-morbidities, mortality was <5% in persons aged ≤80 years.

**Conclusions:**

In this nationwide population-based COVID-19 study, increasing age and multimorbidity were strongly associated with hospitalization and death. In the absence of co-morbidities, the mortality was, however, <5% until the age of 80 years.

**Key Messages**

- We present population-based data on clinical characteristics and predictors of in-patient hospitalization and death for all general-population members who tested positive for SARS-CoV-2 in an entire nation.
- COVID-19 mortality was <5% until the age of 80 years in the absence of co-morbidities, with few patients under the age of 60 years dying regardless of co-morbidities and 79% of fatal cases having at least two co-morbidities.
- A wide range of major chronic diseases were associated with hospitalization and death, with odds ratios ranging from 1.1–1.4 (e.g. stroke, ischaemic heart disease) to 1.8–3.4 (heart failure, hospital-diagnosed kidney disease, chronic liver disease, major psychiatric disorder, organ transplantation).

## Introduction

Despite worldwide efforts to prevent the spread of the severe acute respiratory syndrome coronavirus 2 (SARS-CoV-2), the derived coronavirus disease 2019 (COVID-19) has become a global pandemic. By 22 June 2020, COVID-19 had led to almost 9 000 000 confirmed cases and 470 000 deaths worldwide.^1^ In Denmark, the first COVID-19 case was reported on 27 February 2020 and, after a few weeks, SARS-CoV-2 was widely transmitted in the Danish community.^2^

Hospital-based case series from the early stages of the pandemic have suggested that patients with severe and fatal COVID-19 are likely to be older men with a high burden of co-morbid diseases.^3–7^ Most previous studies were, however, restricted to hospitals and selected populations in areas where the healthcare systems were overwhelmed by the epidemic. Currently, no studies have examined predictors of outcomes in nationwide population-based COVID-19 cohorts in countries with early governmental restrictions and a low burden on the healthcare system. We describe clinical characteristics and predictors of hospitalization and death for all SARS-CoV-2 polymerase chain reaction (PCR)-positive cases in Denmark, where early lockdown and surplus healthcare capacity during the epidemic may have influenced the risk of critical disease.

## Methods

In this population-based study of a Danish COVID-19 cohort capturing all individuals with a positive PCR test for SARS-CoV-2 in Denmark, we provide nationwide data on clinical characteristics and predictors of hospitalization and death for all SARS-CoV-2 PCR-positive cases identified from 27 February 2020 to 19 May 2020. For descriptive comparison, we also provide data on clinical characteristics on all individuals with a negative PCR test for SARS-CoV-2 in Denmark.

### Handling of the epidemic in Denmark

From 27 February 2020 onwards, the spread of SARS-CoV-2 in Denmark was observed within clusters and mainly suspected symptomatic COVID-19 cases with a relevant travel history (mainly from China and Italy) were tested. As of 12 March, community transmission was observed and it was decided to shift from a containment to a mitigation strategy, where testing of patients who had suspected COVID-19 requiring hospital admission was prioritized and contact tracing with quarantine was stopped. The government instituted a comprehensive lockdown of the country on 13 March. On 18 March, testing of frontline healthcare workers in critical functions who had respiratory symptoms was possible and, from late March onwards, test capacity was gradually upscaled to include testing of individuals with mild to moderate respiratory symptoms suspicious of COVID-19, as well as broader screening of healthcare professionals. A controlled and gradual reopening of selected sectors of the country was initiated on 15 April.

### The Danish SARS-CoV-2 cohort

We established the study cohort using data on SARS-CoV-2 PCR results from the Danish Microbiology Database.^8^^,^^9^ Using the unique personal identifier assigned to all Danish citizens, the study cohort was linked to the Danish administrative and health registries.^10–15^ We obtained complete information on the use of prescription drugs filled at community pharmacies, history of hospitalizations and co-morbidities, authorized healthcare-worker status, admission to the intensive care unit (ICU) and date of death, if any (for definitions of variables, see Supplementary Table 1, available as Supplementary data at *IJE* online).

A case was defined as an individual tested one or more times with at least one positive PCR test result for SARS-CoV-2 performed on oro- and nasopharyngeal swabs and/or on respiratory-tract secretions and aspirates. The date of the first positive PCR test was used as the index date whereas individuals with negative SARS-CoV-2 PCR tests were included by the date of their first negative test. Hospital admissions due to COVID-19 were defined as continuous in-hospital stays with a duration of 12 hours or longer occurring up to 14 days after the index date. ICU treatment was defined as intensive-care treatment from 2 days before the index date to 14 days after and was identified using procedure codes in the Danish National Patient Registry^10^ or by direct reporting from the Danish Regions to Statens Serum Institut. All-cause mortality was defined as deaths occurring from 2 days before the index date to 30 days after and identified in the Danish Civil Registration System and the Danish Cause of Death Registry.^11^Real-time data updates and 30 days of follow-up were available for the entire cohort.

### Analysis

We first assessed the number of SARS-CoV-2 PCR-positive cases in Denmark as well as the number of individuals who tested as PCR-negative for SARS-CoV-2. Second, we described clinical characteristics for individuals with negative SARS-CoV-2 PCR results and for PCR-positive cases further stratified by disease severity, i.e. cases who were managed in the community, cases who were hospitalized, cases who were admitted to an ICU and cases who died of all causes within 30 days (inside or outside hospitals). Third, we charted the proportion of hospitalized cases and cases who died of all causes within 30 days, specified by age and assessed predictors of hospitalization and death by estimating crude and age- and sex-adjusted odds ratios (ORs) using logistic regression for the associations between single co-morbidities and hospitalization and death within 30 days. In a post-hoc analysis, predictors of all-cause mortality were assessed in a population restricted to hospitalized SARS-CoV-2 test-positive cases. We chose a logistic regression over a conventional Cox regression as we observed a high number of patients who died very shortly after being tested positive. A survival analysis, like a Cox regression, would put undue emphasis on the time interval between the positive test and death, whereas a logistic regression would merely reflect predictors of whether the patient died or not. To examine whether the outcome associations with age and sex depended on the related burden of co-morbidity, we additionally adjusted age and sex ORs for number of co-morbidities. Co-morbidities were defined as an ever-recording in the Danish health registries of chronic lung disease, hypertension, ischaemic heart disease, heart failure, atrial fibrillation, stroke, diabetes, dementia, any cancer, chronic liver disease, hospital-diagnosed kidney disease, alcohol abuse, substance abuse, major psychiatric disorders, organ transplantation, medical overweight and obesity, and/or rheumatoid arthritis/connective-tissue disease, whereas the number of co-morbidities was defined as the total number of any of these coexisting conditions. In a supplementary analysis, ORs were estimated for each co-morbidity while adjusting for age, sex and additionally for the total number of co-morbidities. Finally, we investigated clinical characteristics of patients diagnosed during different phases of the epidemic in Denmark, defined as the containment phase, the mitigation phase and during the gradual reopening.

### Other

According to Danish law, studies based entirely on registry data do not require patient consent or approval from an ethics review board.^15^ For legal reasons, individual-level raw data from Danish administrative and health registries cannot be shared by the authors.

No patients or members of the public were involved in the creation of this article.

## Results

**Figure 1:**
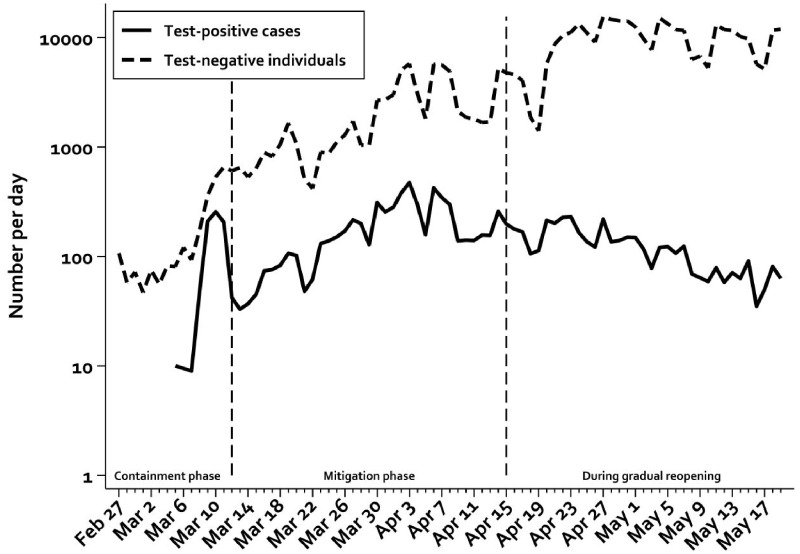
New SARS-CoV-2 PCR-positive cases and the number of individuals who tested negative for SARS-CoV-2 per day during the stages of the ongoing epidemic. The dotted lines illustrate the shift from containment to mitigation strategy as well as to the reopening of society. Note logarithmic *y*-axis. Cases diagnosed before 5 March are omitted to ensure anonymity.

We identified 11 122 cases with SARS-CoV-2 detected by PCR and 410 697 individuals with a negative PCR test in the Danish SARS-CoV-2 cohort from 27 February to 19 May 2020. The number of new SARS-CoV-2 PCR-positive cases peaked at ∼470 cases per day 5–6 weeks after the identification of the first case. The number of new positive cases, however, correlated closely with the number of individuals being tested during the first phases of the epidemic (Figure 1).

In general, we only observed minor differences in age, sex, medical history and prior drug use between PCR-positive cases and test-negative individuals (Table 1). Among all SARS-CoV-2 PCR-positive cases in Denmark, 20% were hospitalized whereas 2.8% were admitted to an ICU and 5.2% had a fatal course of disease within 30 days from the positive test (Table 1). Of those who died, 22% were managed in the community [i.e. did not have an in-hospital admission >12 hours within 14 days after the index date (Supplementary Table 2, available as Supplementary data at *IJE* online)]. The majority of hospitalized cases were admitted on the date of the positive PCR test (57%) (Supplementary Figure 1, available as Supplementary data at *IJE* online). Forty-two per cent of all PCR-positive cases were men, increasing to 73% among cases admitted to an ICU and 57% among cases who died within 30 days of the positive test (Table 1). When adjusted for age and number of co-morbidities, ORs for hospitalization and death were 1.8 [95% confidence interval (CI): 1.6–2.0] and 2.1 (95% CI: 1.7–2.6), respectively, for men (Table 2).

**Table 1:** Baseline characteristics for the overall Danish SARS-CoV-2 cohort and stratified by whether the infection was community-managed or led to any hospitalization, hospitalization without ICU admission, hospitalization with ICU admission or death

	Danish SARS-CoV-2 cohort		SARS-CoV-2 PCR-positive cases				
					Hospitalized		
Characteristic	SARS-CoV-2 test-negative individuals	SARS-CoV-2 PCR-positive cases	Community- managed	Hospitalized	Hospitalized, non-ICU	Hospitalized, ICU	Fatal disease within 30 days^e^
	*n* = 410 697	*n* = 11 122 (100%)	*n* = 8868 (80%)	*n* = 2254 (20%)	*n* = 1940 (17%)	*n* = 314 (2.8%)	*n* = 577 (5.2%)
**Age years, median (IQR)**	46 (30–60)	48 (33–62)	44 (30–56)	71 (56–80)	72 (55–81)	68 (58–75)	82 (75–88)
0–9	23 295 (5.7%)	251 (2.3%)	240 (2.7%)	11 (0.5%)	11 (0.6%)	0 (–)	0 (–)
10–19	25 256 (6.1%)	495 (4.5%)	481 (5.4%)	14 (0.6%)	14 (0.7%)	0 (–)	0 (–)
20–29	52 065 (13%)	1523 (14%)	1468 (17%)	55 (2.4%)	49 (2.5%)	6 (1.9%)	0 (–)
30–39	60 638 (15%)	1588 (14%)	1498 (17%)	90 (4.0%)	80 (4.1%)	10 (3.2%)	16 (2.8%)^**^
40–49	69 424 (17%)	1970 (18%)	1778 (20%)	192 (8.5%)	169 (8.7%)	23 (7.3%)	
50–59	72 603 (18%)	2035 (18%)	1698 (19%)	337 (15%)	285 (15%)	52 (17%)	
60–69	51 445 (13%)	1305 (12%)	931 (10%)	374 (17%)	292 (15%)	82 (26%)	56 (9.7%)
70–79	33 740 (8.2%)	950 (8.5%)	372 (4.2%)	578 (26%)	471 (24%)	107 (34%)	165 (29%)
80–89	17 478 (4.3%)	762 (6.9%)	286 (3.2%)	476 (21%)	442 (23%)	34 (11%)	220 (38%)
90+	4752 (1.2%)	243 (2.2%)	116 (1.3%)	127 (5.6%)	127 (6.5%)	0 (–)	120 (21%)
**Sex**							
Female	253 610 (62%)	6430 (58%)	5388 (61%)	1042 (46%)	956 (49%)	86 (27%)	249 (43%)
Male	157 087 (38%)	4692 (42%)	3480 (39%)	1212 (54%)	984 (51%)	228 (73%)	328 (57%)
**Authorized healthcare workers**	54 004 (13%)	2427 (22%)	2294 (26%)	133 (5.9%)	120 (6.2%)	13 (4.1%)	(*n*<5)
Nurse	20 744 (38%)	1229 (51%)	1173 (51%)	56 (42%)	50 (42%)	6 (46%)	0 (–)
Physician	6335 (12%)	398 (16%)	371 (16%)	27 (20%)	–^***^	(*n* < 5)	(*n* < 5)
Other	26 925 (50%)	800 (33%)	750 (33%)	50 (38%)	–^***^	(*n* < 5)	(*n* < 5)
**Number of co-morbidities** ^a^							
Median (IQR)	0 (0–1)	0 (0–1)	0 (0–1)	2 (1–3)	2 (1–3)	2 (1–3)	3 (2–4)
0	215 795 (53%)	6034 (54%)	5532 (62%)	502 (22%)	433 (22%)	69 (22%)	30 (5.2%)
1	96 736 (24%)	2462 (22%)	1978 (22%)	484 (21%)	412 (21%)	72 (23%)	92 (16%)
2	45 590 (11%)	1140 (10%)	742 (8.4%)	398 (18%)	328 (17%)	70 (22%)	108 (19%)
3	25 312 (6.2%)	691 (6.2%)	327 (3.7%)	364 (16%)	317 (16%)	47 (15%)	122 (21%)
4+	27 264 (6.6%)	795 (7.1%)	289 (3.3%)	506 (22%)	450 (23%)	56 (18%)	225 (39%)
**Hospital admissions within the last year** ^b^							
Median (IQR)	0 (0–0)	0 (0–0)	0 (0–0)	0 (0–1)	0 (0–1)	0 (0–0)	1 (0–2)
0	348 652 (85%)	9597 (86%)	8124 (92%)	1473 (65%)	1235 (64%)	238 (76%)	270 (47%)
1	38 080 (9.3%)	941 (8.5%)	539 (6.1%)	402 (18%)	357 (18%)	45 (14%)	149 (26%)
2	11 766 (2.9%)	269 (2.4%)	107 (1.2%)	162 (7.2%)	150 (7.7%)	12 (3.8%)	71 (12%)
3	5141 (1.3%)	144 (1.3%)	56 (0.6%)	88 (3.9%)	83 (4.3%)	5 (1.6%)	41 (7.1%)
4+	7058 (1.7%)	171 (1.5%)	42 (0.5%)	129 (5.7%)	115 (5.9%)	14 (4.5%)	46 (8.0%)
**Current drug use** ^c^							
Antihypertensive drugs	84 427 (21%)	2389 (21%)	1284 (14%)	1105 (49%)	939 (48%)	166 (53%)	346 (60%)
ACE/ARBs	55 634 (14%)	1589 (14%)	862 (9.7%)	727 (32%)	605 (31%)	122 (39%)	198 (34%)
Calcium channel blockers	31 115 (7.6%)	818 (7.4%)	409 (4.6%)	409 (18%)	346 (18%)	63 (20%)	105 (18%)
Beta-blockers	31 084 (7.6%)	866 (7.8%)	373 (4.2%)	493 (22%)	419 (22%)	74 (24%)	183 (32%)
Thiazides	16 079 (3.9%)	447 (4.0%)	260 (2.9%)	187 (8.3%)	165 (8.5%)	22 (7.0%)	51 (8.8%)
Loop-diuretics	17 420 (4.2%)	580 (5.2%)	178 (2.0%)	402 (18%)	355 (18%)	47 (15%)	195 (34%)
Glucose-lowering drugs	22 137 (5.4%)	755 (6.8%)	388 (4.4%)	367 (16%)	307 (16%)	60 (19%)	110 (19%)
Non-insulin glucose-lowering drugs	17 921 (4.4%)	629 (5.7%)	328 (3.7%)	301 (13%)	254 (13%)	47 (15%)	89 (15%)
Insulin	8009 (2.0%)	286 (2.6%)	124 (1.4%)	162 (7.2%)	140 (7.2%)	22 (7.0%)	58 (10%)
Insulin monotherapy	4216 (1.0%)	126 (1.1%)	60 (0.7%)	66 (2.9%)	53 (2.7%)	13 (4.1%)	21 (3.6%)
Antiplatelets	30 103 (7.3%)	857 (7.7%)	367 (4.1%)	490 (22%)	417 (21%)	73 (23%)	198 (34%)
Anticoagulant therapy	18 098 (4.4%)	577 (5.2%)	234 (2.6%)	343 (15%)	311 (16%)	32 (10%)	163 (28%)
Opioids	32 605 (7.9%)	844 (7.6%)	430 (4.8%)	414 (18%)	376 (19%)	38 (12%)	208 (36%)
Benzodiazepines and derivates	22 027 (5.4%)	538 (4.8%)	280 (3.2%)	258 (11%)	231 (12%)	27 (8.6%)	115 (20%)
Antipsychotics	12 245 (3.0%)	262 (2.4%)	147 (1.7%)	115 (5.1%)	102 (5.3%)	13 (4.1%)	68 (12%)
Antidepressants	41 665 (10%)	996 (9.0%)	604 (6.8%)	392 (17%)	346 (18%)	46 (15%)	167 (29%)
Systemic glucocorticoids	14 579 (3.5%)	344 (3.1%)	151 (1.7%)	193 (8.6%)	170 (8.8%)	23 (7.3%)	78 (14%)
Inhaled corticosteroids	33 717 (8.2%)	760 (6.8%)	453 (5.1%)	307 (14%)	272 (14%)	35 (11%)	89 (15%)
Leukotriene receptor antagonist	3612 (0.9%)	76 (0.7%)	45 (0.5%)	31 (1.4%)	–^***^	(*n* < 5)	(*n* < 5)
Lipid modifying agents	49 864 (12%)	1422 (13%)	761 (8.6%)	661 (29%)	550 (28%)	111 (35%)	191 (33%)
NSAID	46 359 (11%)	1144 (10%)	874 (9.9%)	270 (12%)	220 (11%)	50 (16%)	54 (9.4%)
Methotrexate	1953 (0.5%)	48 (0.4%)	31 (0.3%)	17 (0.8%)	–^***^	(*n* < 5)	6 (1.0%)
Biologics	3380 (0.8%)	65 (0.6%)	52 (0.6%)	13 (0.6%)	–^***^	(*n* < 5)	(*n* < 5)
**Medical history** ^d^							
Chronic lung diseases^*^	58 987 (14%)	1377 (12%)	883 (10.0%)	494 (22%)	433 (22%)	61 (19%)	153 (27%)
Hypertension^*^	93 384 (23%)	2679 (24%)	1448 (16%)	1231 (55%)	1054 (54%)	177 (56%)	413 (72%)
Ischaemic heart disease^*^	29 487 (7.2%)	854 (7.7%)	419 (4.7%)	435 (19%)	377 (19%)	58 (18%)	156 (27%)
Heart failure	9432 (2.3%)	315 (2.8%)	94 (1.1%)	221 (9.8%)	206 (11%)	15 (4.8%)	101 (18%)
Atrial fibrillation	18 768 (4.6%)	603 (5.4%)	249 (2.8%)	354 (16%)	321 (17%)	33 (11%)	170 (29%)
Stroke	16 708 (4.1%)	542 (4.9%)	243 (2.7%)	299 (13%)	269 (14%)	30 (9.6%)	137 (24%)
Diabetes^*^	26 322 (6.4%)	878 (7.9%)	442 (5.0%)	436 (19%)	364 (19%)	72 (23%)	144 (25%)
Dementia^*^	4967 (1.2%)	311 (2.8%)	172 (1.9%)	139 (6.2%)	139 (7.2%)	0 (-)	117 (20%)
Any cancer	31 583 (7.7%)	785 (7.1%)	411 (4.6%)	374 (17%)	317 (16%)	57 (18%)	137 (24%)
Chronic liver disease	6401 (1.6%)	153 (1.4%)	88 (1.0%)	65 (2.9%)	54 (2.8%)	11 (3.5%)	15 (2.6%)
Hospital-diagnosed kidney disease	8538 (2.1%)	294 (2.6%)	100 (1.1%)	194 (8.6%)	172 (8.9%)	22 (7.0%)	80 (14%)
Alcohol abuse^*^	18 799 (4.6%)	298 (2.7%)	184 (2.1%)	114 (5.1%)	99 (5.1%)	15 (4.8%)	37 (6.4%)
Substance abuse^*^	13 515 (3.3%)	185 (1.7%)	128 (1.4%)	57 (2.5%)	–^***^	(*n* < 5)	21 (3.6%)
Major psychiatric disorder^*^	5734 (1.4%)	76 (0.7%)	43 (0.5%)	33 (1.5%)	27 (1.4%)	6 (1.9%)	13 (2.3%)
Organ transplantation	1566 (0.4%)	45 (0.4%)	22 (0.2%)	23 (1.0%)	–^***^	(*n* < 5)	7 (1.2%)
Medical overweight and obesity^*^	38 637 (9.4%)	943 (8.5%)	666 (7.5%)	277 (12%)	239 (12%)	38 (12%)	57 (9.9%)
Rheumatoid arthritis/connective-tissue disease	13 498 (3.3%)	348 (3.1%)	203 (2.3%)	145 (6.4%)	127 (6.5%)	18 (5.7%)	50 (8.7%)

a Number of co-morbidities is the total number of existing conditions listed under ‘Medical history’.

b Hospital admissions of more than 12 hours, from 365 days to 14 days prior to the index date.

c Current drug use is defined as at least one filled prescription within 6 months prior to the test date.

d Medical history is based on an ever-recording of hospital-discharge diagnoses. Co-morbidities marked by ^*^ are defined by hospital-discharge diagnoses in combination with drug use for the co-morbidity (i.e. filled prescription within 6 months prior to the test date). For details on definitions, see Supplementary Table 1, available as Supplementary data at *IJE* online.

e Fatal disease was defined as all-cause mortality within 30 days from the index date.

** Age categories (30–39, 40–49, 50–59 years) collapsed to ensure anonymity.

*** To ensure anonymity, Danish law prohibits reporting of exact *n*= measures (–) in some cases where this could lead to inferring of low *n* results (*n* < 5) in other categories.

ICU, intensive care unit; IQR, interquartile range; SARS-CoV-2, severe acute respiratory syndrome coronavirus 2; ACE, angiotensin-converting enzyme inhibitor; ARB, angiotensin receptor blocker; NSAID, non-steroidal anti-inflammatory drugs.

**Table 2:** Predictors of hospitalization and having a fatal course among SARS-CoV-2 PCR-positive cases

	Hospitalization			**Death within 30 days** ^e^		
Characteristics	Crude OR (95% CI)	Age- and sex-adjusted OR (95% CI)	Age-, sex- and number of co-morbidities-adjusted OR (95% CI)^1^	Crude OR (95% CI)	Age- and sex-adjusted OR (95% CI)	Age-, sex-, and number of co-morbidities-adjusted OR (95% CI)^1^
**Age, years** ^a^						
0–9	0.2 (0.1–0.4)	0.2 (0.1–0.4)	0.3 (0.2–0.6)	NA	NA	NA
10–19	0.1 (0.1–0.3)	0.1 (0.1–0.2)	0.2 (0.1–0.3)	NA	NA	NA
20–29	0.2 (0.1–0.3)	0.2 (0.1–0.3)	0.2 (0.2–0.3)	NA	NA	NA
30–39	0.3 (0.2–0.4)	0.3 (0.2–0.4)	0.4 (0.3–0.5)	NA	NA	NA
40–49	0.5 (0.5–0.7)	0.5 (0.4–0.7)	0.6 (0.5–0.8)	NA	NA	NA
50–59	1.00 (ref.)	1.00 (ref.)	1.00 (ref.)	1.00 (ref.)	1.00 (ref.)	1.00 (ref.)
60–69	2.0 (1.7–2.4)	1.9 (1.6–2.3)	1.6 (1.3–1.9)	6.0 (3.4–10.7)	5.6 (3.2–10.0)	4.4 (2.5–7.9)
70–79	7.8 (6.6–9.3)	7.5 (6.2–8.9)	4.7 (3.9–5.7)	28.3 (16.6–48.3)	25.9 (15.2–44.3)	15.2 (8.7–26.3)
80–89	8.4 (7.0–10.1)	8.6 (7.1–10.3)	4.8 (3.9–5.8)	54.7 (32.1–93.0)	55.6 (32.7–94.8)	29.9 (17.2–51.9)
90+	5.5 (4.2–7.3)	6.1 (4.6–8.1)	3.5 (2.6–4.7)	131.4 (74.5–231.6)	155.2 (87.6–275.1)	90.2 (50.2–162.2)
**Sex** ^a^						
Female	1.00 (ref.)	1.00 (ref.)	1.00 (ref.)	1.00 (ref.)	1.00 (ref.)	1.00 (ref.)
Male	1.8 (1.6–2.0)	1.8 (1.6–2.0)	1.8 (1.6–2.0)	1.9 (1.6–2.2)	2.1 (1.7–2.6)	2.1 (1.7–2.6)
**Authorized healthcare workers**						
Non-healthcare worker	1.00 (ref.)	1.00 (ref.)	1.00 (ref.)	1.00 (ref.)	1.00 (ref.)	1.00 (ref.)
Nurse	0.1 (0.1–0.2)	0.4 (0.3–0.5)	0.4 (0.3–0.6)	NA	NA	NA
Physician	0.2 (0.2–0.3)	0.5 (0.3–0.7)	0.5 (0.3–0.8)	NA	NA	NA
Other	0.2 (0.2–0.3)	0.5 (0.3–0.6)	0.5 (0.4–0.6)	NA	NA	NA
**Number of co-morbidities** ^b^						
0	1.00 (ref.)	1.00 (ref.)		1.00 (ref.)	1.00 (ref.)	
1	2.7 (2.4–3.1)	1.7 (1.5–2.0)		7.8 (5.1–11.8)	2.6 (1.6–4.0)	
2	5.9 (5.1–6.9)	2.1 (1.8–2.5)		20.9 (13.9–31.6)	2.6 (1.7–4.1)	
3	12.3 (10.3–14.6)	3.1 (2.5–3.8)		42.9 (28.5–64.6)	3.5 (2.2–5.4)	
4+	19.3 (16.3–22.9)	3.9 (3.2–4.8)		79.0 (53.5–116.7)	5.2 (3.4–8.0)	
**Hospital admissions within the last year** ^c^						
0	1.00 (ref.)	1.00 (ref.)	1.00 (ref.)	1.00 (ref.)	1.00 (ref.)	1.00 (ref.)
1	4.1 (3.6–4.7)	2.2 (1.9–2.7)	1.9 (1.6–2.3)	6.5 (5.3–8.0)	2.1 (1.6–2.7)	1.8 (1.4–2.4)
2	8.4 (6.5–10.7)	3.6 (2.7–4.8)	3.0 (2.2–4.0)	12.4 (9.2–16.7)	3.2 (2.3–4.5)	2.8 (1.9–3.9)
3	8.7 (6.2–12.2)	2.7 (1.9–4.0)	2.1 (1.4–3.1)	13.8 (9.4–20.1)	2.7 (1.8–4.2)	2.1 (1.4–3.3)
4+	16.9 (11.9–24.1)	7.2 (4.8–10.7)	5.0 (3.3–7.5)	12.7 (8.9–18.2)	3.1 (2.0–4.6)	2.3 (1.5–3.4)
**Medical history** ^d^						
Chronic lung diseases^*^	2.5 (2.2–2.9)	1.8 (1.5–2.1)		2.7 (2.3–3.3)	1.4 (1.1–1.8)	
Hypertension^*^	6.2 (5.6–6.8)	1.7 (1.5–1.9)		9.2 (7.6–11.1)	1.3 (1.1–1.6)	
Ischaemic heart disease^*^	4.8 (4.2–5.6)	1.4 (1.2–1.7)		5.2 (4.3–6.4)	1.1 (0.9–1.4)	
Heart failure	10.1 (7.9–13.0)	2.6 (2.0–3.4)		10.2 (7.9–13.2)	1.8 (1.3–2.4)	
Loop-diuretic use^**^	10.6 (8.8–12.7)	2.5 (2.1–3.1)		13.5 (11.0–16.5)	2.2 (1.7–2.7)	
Atrial fibrillation	6.4 (5.4–7.6)	1.4 (1.2–1.7)		9.8 (8.0–12.0)	1.6 (1.2–2.0)	
Stroke	5.4 (4.6–6.5)	1.3 (1.1–1.6)		7.8 (6.3–9.7)	1.4 (1.1–1.8)	
Diabetes^*^	4.6 (4.0–5.3)	1.8 (1.6–2.2)		4.4 (3.6–5.4)	1.6 (1.3–2.0)	
Non-insulin glucose-lowering-drug use^**^	4.0 (3.4–4.7)	1.7 (1.4–2.1)		3.4 (2.7–4.3)	1.3 (1.0–1.8)	
Any insulin use^**^	5.5 (4.3–6.9)	2.3 (1.7–3.0)		5.1 (3.7–6.8)	1.9 (1.3–2.6)	
Insulin monotherapy use^**^	4.4 (3.1–6.3)	2.4 (1.5–3.6)		3.8 (2.3–6.0)	1.5 (0.9–2.6)	
Dementia^*^	3.3 (2.6–4.2)	0.5 (0.4–0.7)		13.6 (10.6–17.4)	2.0 (1.5–2.6)	
Any cancer	4.1 (3.5–4.7)	1.4 (1.2–1.6)		4.8 (3.9–5.9)	1.3 (1.0–1.7)	
Chronic liver disease	3.0 (2.1–4.1)	2.3 (1.6–3.3)		2.0 (1.2–3.5)	1.8 (1.0–3.3)	
Hospital-diagnosed kidney disease	8.3 (6.5–10.6)	2.9 (2.2–3.9)		7.8 (5.9–10.2)	1.9 (1.4–2.6)	
Alcohol abuse^*^	2.5 (2.0–3.2)	1.7 (1.3–2.3)		2.7 (1.9–3.9)	1.8 (1.2–2.7)	
Substance abuse^*^	1.8 (1.3–2.4)	1.3 (0.9–1.9)		2.4 (1.5–3.8)	1.8 (1.1–3.2)	
Major psychiatric disorder^*^	3.0 (1.9–4.8)	2.1 (1.2–3.7)		3.8 (2.1–7.0)	2.5 (1.2–5.1)	
Benzodiazepines and derivates use^**^	4.0 (3.3–4.7)	1.7 (1.4–2.1)		6.0 (4.8–7.5)	2.0 (1.6–2.6)	
Antipsychotic use^**^	3.2 (2.5–4.1)	1.5 (1.1–1.9)		7.1 (5.3–9.5)	3.3 (2.3–4.8)	
Antidepressant use^**^	2.9 (2.5–3.3)	1.3 (1.1–1.5)		4.8 (3.9–5.8)	1.7 (1.3–2.1)	
Organ transplantation	4.1 (2.3–7.5)	3.4 (1.7–6.6)		3.4 (1.5–7.6)	3.2 (1.3–8.4)	
Medical overweight and obesity^*^	1.7 (1.5–2.0)	2.1 (1.8–2.5)		1.2 (0.9–1.6)	1.5 (1.1–2.0)	
Rheumatoid arthritis/connective-tissue disease	2.9 (2.4–3.7)	1.5 (1.1–1.9)		3.3 (2.4–4.5)	1.1 (0.8–1.6)	

a Age was adjusted for sex and number of co-morbidities whereas sex was adjusted for age and number of co-morbidities.

b Number of co-morbidities is the total number of coexisting conditions listed under ‘Medical history’.

c Hospital admissions of more than 12 hours, from 365 days to 14 days prior to the index date.

d Medical history is based on an ever-recording of hospital-discharge diagnoses. Co-morbidities marked by * are defined by hospital-discharge diagnoses in combination with drug use for the co-morbidity (i.e. filled prescription within 6 months prior to the test date). ** denotes exclusive use of drugs that are close markers of specific underlying co-morbidities, assessed independently of the presence or absence of hospital diagnoses for the co-morbidity. For details on definitions, see Supplementary Table 1, available as Supplementary data at *IJE* online.

e Death was defined as all-cause mortality within 30 days from the index date.

OR, odds ratio; SARS-CoV-2, severe acute respiratory syndrome coronavirus 2; NA, not applicable due to too few cases.

Grey boxes for ‘Medical history’ indicate that ORs for single co-morbidities were not adjusted for total number of co-morbidities in the main analysis, because some co-morbidities may be an effect of the index co-morbidity. For further exploratory analyses and details, see Supplementary Table 4, available as Supplementary data at *IJE* online.

**Figure 2:**
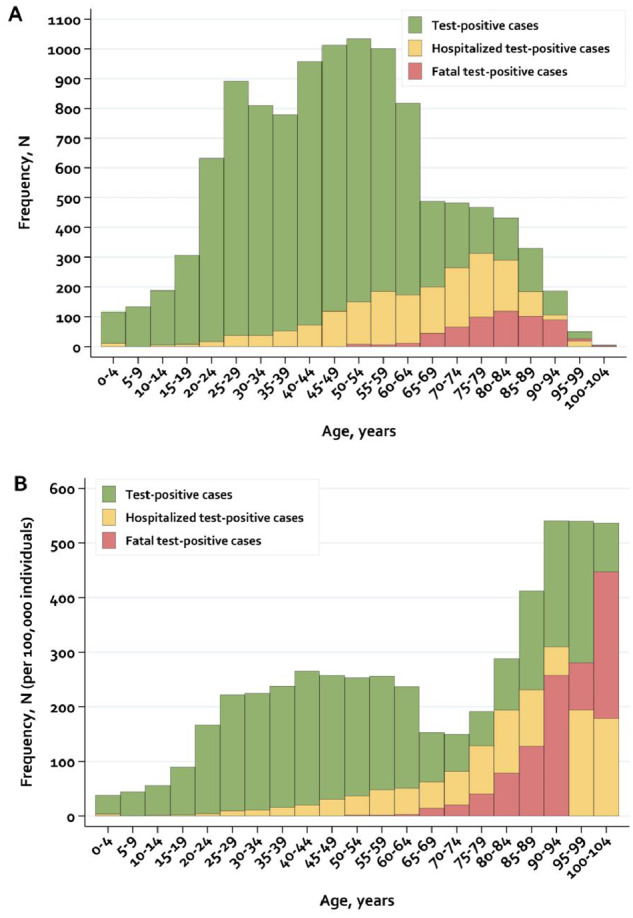
(A) Distribution of hospitalization and death according to age group in all SARS-CoV-2 PCR-positive cases. (B) The proportion of hospitalized and fatal SARS-CoV-2 cases per 100 000 individuals relative to the total Danish population within each age group Fatal cases were defined as all PCR-positive cases who died of all causes within 30 days from the index date.

Among all PCR-positive cases, the median age was 48 years [interquartile range (IQR) 33–62], varying from 44 years (IQR 30–56) among cases who were not hospitalized to 82 years (IQR 75–88) among those who died (Table 1). The proportion of SARS-CoV-2 PCR-positive cases who were hospitalized increased substantially with age to >60% among cases older than 70 years. Similarly, the proportion of PCR-positive cases with a fatal course increased from 17% by the age of 70–79 years to 29% by the age of 80–89 years (Figure 2A). When applied to the number of individuals in the Danish population in each age group, population mortality likewise increased from 29 per 100 000 for individuals aged 70–70 years to 266 per 100 000 by the age of 90 years (Figure 2B). When adjusting for sex and number of co-morbidities, increasing age was a very strong predictor of fatal disease [OR 15 (95% CI: 9–26) for 70–79 years, OR 30 (95% CI: 17–52) for 80–89 years and OR 90 (95% CI: 50–162) for cases older than 90 years, when compared with middle-aged adults, 50–59 years] (Table 2).

In general, co-morbidities were more frequent among PCR-positive hospitalized and fatal cases. Thus, 15% of community-managed cases had two or more co-morbidities, whereas the corresponding proportion was 56% for hospitalized and 79% for fatal cases. Similarly, the proportion of individuals who had been hospitalized at least once during the last year was higher among hospitalized cases (35%) and fatal cases (53%) than among PCR-positive cases managed in the community (8%) (Table 1).

The most frequent co-morbidities among hospitalized cases were hypertension (55%), COPD (22%), ischaemic heart disease (19%) and diabetes (19%) (Table **1**). After adjustment for higher age and sex, the association of many co-morbidities with hospitalization risk reduced considerably (e.g. for dementia, from OR 3.3 to OR 0.5). However, most co-morbidities remained predictive of COVID-19 hospitalization, ranging from OR 1.3 for stroke, OR 1.4 for atrial fibrillation, cancer or ischaemic heart disease, to OR 1.7 for hypertension or alcohol abuse, OR 1.8 for chronic lung disease or diabetes, and peaking at OR 2.9 for hospital-diagnosed kidney disease and OR 3.4 for organ transplantation (Table 2). This pattern was also evident among fatal cases, though the absolute prevalence of co-morbidities was higher in fatal than in hospitalized cases (Tables 1 and 2). Among PCR-positive cases with four or more co-morbidities, the ORs for hospitalization were 3.9 (95% CI: 3.2–4.8) and 5.2 (95% CI: 3.4–8.0) for death compared with PCR-positive cases without any co-morbidities (Table 2). When restricting the population to hospitalized cases (Supplementary Table 3, available as Supplementary data at *IJE* online), largely similar results were observed.

**Figure 3.**
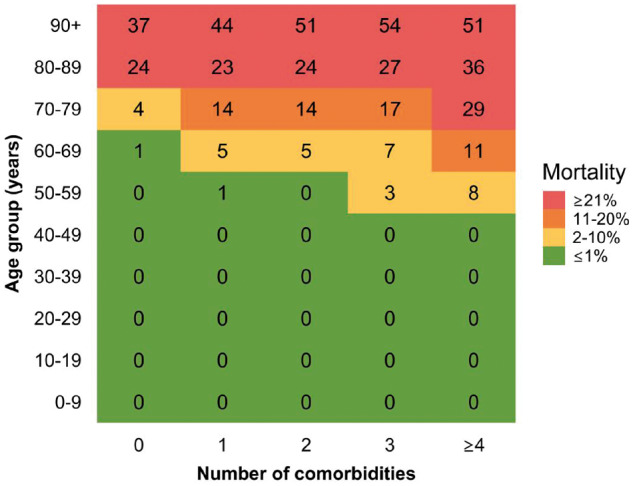
Heat map illustrating proportion of patients dying (in %) among SARS-CoV-2 PCR-positive cases within different subgroups of age and number of co-morbidities Total number of co-morbidities is assessed as the total number of any of the following conditions: chronic lung disease, hypertension, ischaemic heart disease, heart failure, atrial fibrillation, stroke, diabetes, dementia, cancer, chronic liver disease, hospital-diagnosed kidney disease, alcohol abuse, substance abuse, major psychiatric disorder, organ transplantation, overweight and obesity, and rheumatoid arthritis/connective-tissue disease. Mortality was defined as all-cause mortality within 30 days from the index date. Stated mortality proportions in % in each subgroup are rounded to the nearest number (green <1.50%; yellow 1.50–10.49%; orange 10.50–20.49%; red >20.49%). The value 0% means that <0.5% in the subgroup died or that there were no patients in the subgroup.

All-cause mortality increased substantially with increasing age in combination with an increasing number of co-morbidities (Figure 3). Among cases aged 60–69 and 70–79 years with no co-morbidities, the mortality was 1% and 4%, respectively, although increasing to 11% and 29%, respectively, among those with at least four co-morbidities. Among the highest age categories, mortality was high regardless of the number of co-morbidities (Figure 3).

When further adjusting for the total number of co-morbidities, the ORs for patients with individual co-morbidities declined considerably (Supplementary Table 4, available as Supplementary data at *IJE* online), suggesting that multimorbidity and frailty in patients with e.g. hypertension, diabetes or cardiopulmonary disease may be a key driver of the observed associations.

Authorized healthcare workers comprised 22% of all PCR-positive cases (Table 1). Of these, 133 cases (5.5%) were hospitalized, 13 cases (0.5%) required ICU admission and <5 cases (<0.2%) died within 30 days of the positive test.

Patient characteristics of all PCR-positive cases changed markedly during the different stages of the epidemic. Thus, the proportion of women increased from 32% in the initial stage [when travellers from risk areas (often males) were frequently tested] to 60% during the reopening stage [when healthcare workers (of whom 89% were females in our study population) were frequently tested]. Mortality (6.7%) and median age (52 years, IQR 38–66) were highest during the mitigation phase where predominantly individuals who were hospitalized were tested (Supplementary Table 5, available as Supplementary data at *IJE* online).

## Discussion

In this nationwide cohort of SARS-CoV-2 PCR-positive cases from the general population in Denmark, we found that older age (e.g. >70 years), male sex and number of co-morbidities were risk factors for hospitalization and death. In the absence of co-morbidities, the mortality was, however, <5% until the age of 80 years. After controlling for age and sex, virtually all co-morbidities that were prevalent in our population, including e.g. hypertension, heart or lung disease, obesity and diabetes were associated with severe disease or death from COVID-19. Particularly strong associations were observed for hospital-diagnosed kidney disease, severe psychiatric disorder and organ transplantation.

The register-based approach and Denmark’s universal healthcare system are major strengths of this study, since the Danish administrative and health registries allow complete nationwide capture of an unselected cohort of all individuals tested for SARS-CoV-2 without restricting to those treated at hospitals and irrespective of socio-economic differences. Population-based registries allowed complete, independent individual-level ascertainment of all previous hospital contacts and prescription-drug use, overcoming limitations of missing data in previous reports. It is, however, a limitation that co-morbidities often followed in primary care, e.g. obesity and dementia, may be underreported in the Danish hospital and prescription registries, thus causing an underestimation of the prevalence of these specific diseases. It is also a limitation that we did not have information on several factors such as smoking status, ethnicity and socio-economic deprivation that are likely to influence the risk of developing severe COVID-19 or dying.^16^^,^^17^ Finally, we made no attempts to determine the fraction of deaths directly attributable to COVID-19 as this is subjective and difficult using registry data. The use of 30-day all-cause mortality in our study may have led to age associations appearing more extreme.

The existence of associations between almost any co-morbidities and the risk of hospitalization and death due to COVID-19 in our study is in accordance with previous studies of both COVID-19 patients^18^^,^^19^ and patients with severe influenza,^20^ thus suggesting resemblances between COVID-19 and other severe respiratory infections with regard to populations at risk. Among the most frequent co-morbidities in our population, hypertension, obesity and diabetes seemed to be clear predictors of both hospitalization and fatal disease, which corroborates previous hospital-based outcome studies^21^^,^^22^ and underscores the probable importance of metabolic health in COVID-19 outcomes.^23^ Our data add important new knowledge on the possible role of hospital-diagnosed kidney disease and organ transplantation as strong risk factors, and furthermore suggest that people with alcohol/substance abuse and psychiatric illness may be an especially vulnerable group, possibly in line with the socio-economic disparities that have been observed during the COVID-19 epidemic.^16^^,^^17^

Importantly, the assessment of predictors in our study was performed without having any pre-specified hypotheses. We did not aim to estimate causal effects, but rather to identify factors that could help us to identify people at high risk of hospitalization and all-cause mortality during COVID-19. The potential causal associations of specific individual diseases with COVID-19 outcomes, including the potentially strong association observed for e.g. metabolic diseases, should be analysed in future epidemiological studies designed to evaluate causal effects, including detailed, hypothesis-specific confounder assessment. Any associations observed in this study should therefore be interpreted with caution and not as evidence of causality. Moreover, the threshold for diagnosing co-morbidities as well as COVID-19 may differ across age groups. Among elderly multimorbid persons, Berksonian-like bias may have caused an overestimation of COVID-19-outcome associations if some hospital admissions were primarily related to worsening underlying co-morbidities rather than infection, and then led to testing and coincident detection of SARS-CoV-2.

It is of note that the different test strategies instituted in Denmark during the epidemic are crucial for the observed characteristics of individuals with confirmed SARS-CoV-2 infection. In the early stages of the pandemic, the national test strategy in Denmark was directed at those who were most sick and potentially in need of medical care. Though the observed overall 30-day mortality rate of 5.2% observed in our study corresponds to the case-fatality rate observed worldwide (5.1%) (https://www.worldometers.info/coronavirus/), the fact that mainly hospitalized patients with severe symptoms were tested for SARS-CoV-2 during the mitigation phase may have contributed to an overestimation of the case fatality and the proportion of hospitalized cases (20%) in COVID-19. Preliminary results from serologic (antibody-testing) studies in Denmark suggest that, in May 2020, 1.1% (municipality screening, www.ssi.dk) to 1.8% (blood-donor screening, unpublished data) of all Danish citizens had been infected with SARS-CoV-2, corresponding to an estimated 64 000 to 105 000 COVID-19 patients in Denmark (population 5.82 million). This suggests that the prevalence of SARS-CoV-2 infection may have been 6- to 9-fold higher than detected in our study, thus corresponding to a possible infection fatality rate of 0.6–0.9% rather than the 5.2% that we observed.

Also, the high absolute number of PCR-positive healthcare professionals may reflect widespread testing in this group to track down and limit in-hospital contamination, rather than a particularly high level of contamination of healthcare professionals. Widespread testing with detection of asymptomatic or mild symptomatic cases in this group may also play a role for the observed lower risk of hospital admission or death. Furthermore, the source of transmission is unknown. It is a limitation in our study that the subgroup of healthcare professionals only included individuals authorized as healthcare personnel, i.e. mainly nurses and doctors, but not professions for which an authorization is not required, e.g. hospital porters and nurse assistants. Further data on whether the healthcare professionals were involved in clinical work or working in other settings were not available, thus some healthcare professionals without current patient contact were included.

In accordance with previous descriptive studies, older men with co-morbidities dominated the subgroup of cases with severe or fatal disease.^24^^,^^25^ Additionally, we found that men had a 2.1-fold risk of death even when adjusting for age and number of co-morbidities, thus suggesting that the higher risk of severe or fatal disease among men cannot be explained by a higher burden of co-morbidities in its entirety. In our study, cases admitted to an ICU were markedly younger and less co-morbid than cases with a fatal course. This may be related to selection of those patients for the ICU who are expected to clearly gain from intensive-care therapy when it comes to later life expectancy, functional status and quality of life. Some very elderly and frail patients may have passed away more rapidly upon hospitalization and thus never have been transferred to the ICU. Also, COVID-19 cases with a fatal course who were not hospitalized (22% of fatalities) were older and more likely to have dementia than those who died following hospitalization. This finding is likely caused by smaller outbreaks at nursing homes and suggests that frailty impacted on the clinical decision of whether to admit COVID-19 cases or not. Of note, the median age of 82 years at death from COVID-19 in our study is almost identical to the median age at death (81 years) of general-population members in Denmark.^26^

The findings of this study should be seen from the perspective that the COVID pandemic in Denmark has been characterized by a strict, early lockdown and a rapid adaptation of healthcare to the situation. When the pandemic peaked in the beginning of April, 142 patients were admitted to ICUs nationwide.^27^ With a reported capacity of 925 mechanical ventilators available for COVID-19 patients,^28^ the healthcare system was never overwhelmed. Also, the early lockdown may have emphasized the seriousness of the pandemic in the public at large, prompting patients to report early in their disease course and hospitals to admit COVID-19 patients at a lower threshold. Easy access and affordability of healthcare could play a similar role.

## Conclusion

In this nationwide population-based study, increasing age, sex and the number and type of co-morbidities were closely associated with hospitalization and death in SARS-CoV-2 PCR-positive cases. In the absence of co-morbidities, the mortality was, however, lowest until the age of 80 years. These results may help in accurate identification, triage and protection of high-risk groups in general populations, i.e. when reopening societies.

## Supplementary data

Supplementary data are available at *IJE* online.

## Author contributions

All authors designed the study, interpreted the data, revised the manuscript and approved the final version of the manuscript. M.T.E. cleaned and analysed the data. K.B.K. validated the code used for data cleaning and analysis.

## Supplementary Material

dyaa140_supplementary_data
