# Supplementary material for: Characteristics and predictors of hospitalization and death in the first 11 122 cases with a positive RT-PCR test for SARS-CoV-2 in Denmark: a nationwide cohort
Source: Int J Epidemiol. 2020 Sep 5;49(5):1468–81. doi: 10.1093/ije/dyaa140 (PMC7499657; doi:10.1093/ije/dyaa140)

**Supplementary**

**Supplementary Table S1.** ICD-10- and ATC-codes used to define drug and comorbidity.

|  | Coding system | Codes |
| --- | --- | --- |
| *Current drug use^1^* |  |  |
| Antihypertensive drugs | ATC | C03A C07 C08 C09 |
| ACE/ARBs | ATC | C09 |
| Calcium channel blockers | ATC | C08 |
| Beta-blockers | ATC | C07 |
| Thiazides | ATC | C03A |
| Loop-diuretics | ATC | C03C |
| Glucose-lowering drugs | ATC | A10A A10B |
| Non-insulin glucose lowering drugs | ATC | A10B |
| Insulin | ATC | A10A |
| Insulin monotherapy | ATC | A10A, not A10B |
| Antiplatelets | ATC | B01AC |
| Anticoagulant therapy | ATC | B01AA, B01AE07, B01AF |
| Opioids | ATC | N02A |
| Benzodiazepines and derivates | ATC | N05BA N05CD-F |
| Antipsychotics | ATC | N05AA N05AB N05AC N05AD N05AE N05AF N05AG N05AH N05AX N05AL01 N05AL05 |
| Antidepressants | ATC | N06A |
| Systemic glucocorticoids | ATC | H02AB |
| Inhaled corticosteroids | ATC | R03AK R03AL R03BA |
| Lipid modifying agents | ATC | C10 |
| NSAIDs | ATC | M01A (excluding M01AX) |
| Methotrexate | ATC | L04AX03 |
| Biologics | SKS | BOHJ16A BOHJ18A1-5 BOHJ18B1-8 BOHJ18C1 BOHJ19H4 BOHJ19H6 BOHJ26 BWHB84 |
|  | ATC | L04AA21 L04AA23-6 L04AA28 L04AA33  L04AA34 L04AA36 L04AB01 L04AB02 L04AB04 L04AB05 L04AC02 L04AC03 L04AC05 L04AC07 L04AC08 L04AC10-4 L04AC16 L04AC17  D11AH05 L01XC02 |
| *Medical history^2^* |  |  |
| Chronic lung disease | ICD-10 | J41-J47 |
|  | ATC | R03AK, R03AL, R03BA, R03AC12, R03AC13, R03AC18, R03AC19, R03CC12, R03BB04, R03BB05, R03BB06, R03BB07 |
| Hypertension | ICD-10 | I10 I11 I12 I13 I15 |
|  | ATC | C08, C03A, C07, C09 |
| Ischemic heart disease | ICD-10 | I20 I21 I22 I23 I24 I25 |
|  | ATC | N02BA C01DA B01AC24 |
| Heart failure | ICD-10 | I099A I110 I130 I132 I50 |
| Atrial fibrillation | ICD-10 | I48 |
| Stroke | ICD-10 | I60 I61 I62 I63 I64 I69 |
| Diabetes | ICD-10 | E10 E11 E13 E14 |
|  | ATC | A10 |
| Dementia | ICD-10 | F00 F01 F02 F03 F1073 F1173 F1273 F1373 F1473 F1573 F1673 F1873 F1973 |
|  | ATC | N06D |
| Any cancer | ICD-10 | C00-C97, excluding C44 |
| Chronic liver disease | ICD-10 | K700-K704 K709 K71-K74 K760 K766 B150 B160 B162 B18 B190 I85 |
| Hospital-diagnosed kidney disease | ICD-10 | I12 I13 N00-N05 N07 N08 N11 N14 N18 N19 E102 E112 E142 |
| Alcohol abuse | ICD-10 | F10 E244 G312 G621 G721 I426 K292 K70 K852 K860 Q860 Z502 Z714 Z721 |
|  | ATC | N07BB |
| Substance abuse | ICD-10 | F11-F19 |
|  | ATC | N07BC |
| Organ transplantation | ICD-10 | Z94 |
| Medical overweight and obesity | ICD-10 | E66 |
|  | ATC | A08 |
| Severe mental illness (schizophrenia, schizoaffective disorder, or bipolar disorder) | ICD-10 | F20 F25 F30 F31 |
|  | ATC | N05AN |

^1^Current drug use is defined as at least one filled prescription within 6 months prior to the test date.

^2^Medical history is based on an ever-recording of hospital discharge diagnoses, with or without combination with drug redemption data.

ACE: angiotensin-converting enzyme inhibitor; ARB: angiotensin receptor blocker; NSAID: non-steroidal anti-inflammatory drugs.

**Supplementary Table S2.** Baseline characteristics for SARS-CoV-2 PCR-positive cases with a fatal course within 30 days, stratified by whether cases were managed in the community or hospitalized prior to their death.

|  | **SARS-CoV-2 PCR-positive cases with fatal course** | | | | |
| --- | --- | --- | --- | --- | --- |
|  |  | All | | Hospitalized | |
| Characteristic | All | Community-managed** | Hospitalized | Non-ICU admission | ICU admission |
|  | n=577 (100%) | n=127 (22%) | n=450 (78%) | n=364 (63%) | n=86 (15%) |
| **Age years, median (IQR)** | 82 (75-88) | 87 (78-91) | 81 (74-86) | 82 (76-88) | 74 (69-77) |
| 0-29 | 0 (-)*** | 0 (-)*** | 0 (-)*** | 0 (-)*** | 0 (-)*** |
| 30-69 | 72 (12%) | 8 (6.3%) | 64(14%) | 39 (11%) | 25 (29%) |
| 70-79 | 165 (29%) | 27 (21%) | 138 (31%) | 93 (26%) | 45 (52%) |
| 80-89 | 220 (38%) | 46 (36%) | 174 (39%) | 158 (43%) | 16 (19%) |
| 90+ | 120 (21%) | 46 (36%) | 74 (16%) | 74 (20%) | 0 (-) |
| **Sex** |  |  |  |  |  |
| Female | 249 (43%) | 76 (60%) | 173 (38%) | 157 (43%) | 16 (19%) |
| Male | 328 (57%) | 51 (40%) | 277 (62%) | 207 (57%) | 70 (81%) |
| **Authorized health care workers** | (n<5) | 0 (-) | (n<5) | (n<5) | (n<5) |
| Nurse | 0 (-) | 0 (.%) | 0 (-) | 0 (-) | 0 (-) |
| Physician | (n<5) | 0 (.%) | (n<5) | 0 (-) | (n<5) |
| Other | (n<5) | 0 (.%) | (n<5) | (n<5) | (n<5) |
| **Number of comorbidities^1^** |  |  |  |  |  |
| Median [IQR] | 3 (2-4) | 3 (1-4) | 3 (2-4) | 3 (2-4) | 2 (1-4) |
| 0 | 30 (5.2%) | 9 (7.1%) | 21 (4.7%) | 15 (4.1%) | 6 (7.0%) |
| 1 | 92 (16%) | 24 (19%) | 68 (15%) | 48 (13%) | 20 (23%) |
| 2 | 108 (19%) | 28 (22%) | 80 (18%) | 60 (16%) | 20 (23%) |
| 3 | 122 (21%) | 22 (17%) | 100 (22%) | 84 (23%) | 16 (19%) |
| 4+ | 225 (39%) | 44 (35%) | 181 (40%) | 157 (43%) | 24 (28%) |
| **Hospital admissions within the last year^2^** |  |  |  |  |  |
| Median [IQR] | 1 (0-2) | 1 (0-1) | 1 (0-2) | 1 (0-2) | 0 (0-1) |
| 0 | 270 (47%) | 63 (50%) | 207 (46%) | 157 (43%) | 50 (58%) |
| 1 | 149 (26%) | 36 (28%) | 113 (25%) | 97 (27%) | 16 (19%) |
| 2 | 71 (12%) | 14 (11%) | 57 (13%) | 47 (13%) | 10 (12%) |
| 3+ | 87 (15%) | 14 (11%) | 73 (16%) | 63 (17%) | 10 (12%) |
| **Current drug use^3^** |  |  |  |  |  |
| Antihypertensive drugs | 346 (60%) | 57 (45%) | 289 (64%) | 238 (65%) | 51 (59%) |
| ACE/ARBs | 198 (34%) | 28 (22%) | 170 (38%) | 135 (37%) | 35 (41%) |
| Calcium channel blockers | 105 (18%) | 12 (9.4%) | 93 (21%) | 78 (21%) | 15 (17%) |
| Beta-blockers | 183 (32%) | 25 (20%) | 158 (35%) | 132 (36%) | 26 (30%) |
| Thiazides | 51 (8.8%) | 13 (10%) | 38 (8.4%) | 30 (8.2%) | 8 (9.3%) |
| Loop-diuretics | 195 (34%) | 38 (30%) | 157 (35%) | 135 (37%) | 22 (26%) |
| Glucose-lowering drugs | 110 (19%) | 16 (13%) | 94 (21%) | 74 (20%) | 20 (23%) |
| Non-insulin glucose lowering drugs | 89 (15%) | 11 (8.7%) | 78 (17%) | 60 (16%) | 18 (21%) |
| Insulin | 58 (10%) | 8 (6.3%) | 50 (11%) | 43 (12%) | 7 (8.1%) |
| Insulin monotherapy | 21 (3.6%) | 5 (3.9%) | 16 (3.6%) | -**** | (n<5) |
| Antiplatelets | 198 (34%) | 35 (28%) | 163 (36%) | 131 (36%) | 32 (37%) |
| Anticoagulant therapy | 163 (28%) | 33 (26%) | 130 (29%) | 113 (31%) | 17 (20%) |
| Opioids | 208 (36%) | 56 (44%) | 152 (34%) | 132 (36%) | 20 (23%) |
| Benzodiazepines and derivates | 115 (20%) | 32 (25%) | 83 (18%) | 74 (20%) | 9 (10%) |
| Antipsychotics | 68 (12%) | 23 (18%) | 45 (10%) | -**** | (n<5) |
| Antidepressants | 167 (29%) | 54 (43%) | 113 (25%) | 100 (27%) | 13 (15%) |
| Systemic glucocorticoids | 78 (14%) | 17 (13%) | 61 (14%) | 54 (15%) | 7 (8.1%) |
| Inhaled corticosteroids | 89 (15%) | 15 (12%) | 74 (16%) | 64 (18%) | 10 (12%) |
| Leukotriene receptor antagonist | (n<5) | (n<5) | (n<5) | (n<5) | (n<5) |
| Lipid modifying agents | 191 (33%) | 29 (23%) | 162 (36%) | 126 (35%) | 36 (42%) |
| NSAID | 54 (9.4%) | (n<5) | -**** | 35 (9.6%) | 16 (19%) |
| Methotrexate | 6 (1.0%) | 0 (-) | 6 (1.3%) | (n<5) | (n<5) |
| Biologics | (n<5) | 0 (-) | (n<5) | (n<5) | 0 (-) |
| **Medical history^4^** |  |  |  |  |  |
| Chronic lung diseases* | 153 (27%) | 25 (20%) | 128 (28%) | 104 (29%) | 24 (28%) |
| Hypertension* | 413 (72%) | 82 (65%) | 331 (74%) | 277 (76%) | 54 (63%) |
| Ischemic heart disease* | 156 (27%) | 33 (26%) | 123 (27%) | 102 (28%) | 21 (24%) |
| Heart failure | 101 (18%) | 19 (15%) | 82 (18%) | 74 (20%) | 8 (9.3%) |
| Atrial fibrillation | 170 (29%) | 38 (30%) | 132 (29%) | 120 (33%) | 12 (14%) |
| Stroke | 137 (24%) | 35 (28%) | 102 (23%) | 91 (25%) | 11 (13%) |
| Diabetes* | 144 (25%) | 25 (20%) | 119 (26%) | 95 (26%) | 24 (28%) |
| Dementia* | 117 (20%) | 42 (33%) | 75 (17%) | 75 (21%) | 0 (-) |
| Any cancer | 137 (24%) | 27 (21%) | 110 (24%) | 89 (24%) | 21 (24%) |
| Chronic liver disease | 15 (2.6%) | (n<5) | -**** | -**** | (n<5) |
| Hospital-diagnosed kidney disease | 80 (14%) | 9 (7.1%) | 71 (16%) | 60 (16%) | 11 (13%) |
| Alcohol abuse* | 37 (6.4%) | 6 (4.7%) | 31 (6.9%) | 25 (6.9%) | 6 (7.0%) |
| Substance abuse* | 21 (3.6%) | (n<5) | -**** | 16 (4.4%) | (n<5) |
| Major psychiatric disorder | 13 (2.3%) | (n<5) | -**** | -**** | (n<5) |
| Organ transplantation | 7 (1.2%) | 0 (-) | 7 (1.6%) | (n<5) | (n<5) |
| Medical overweight and obesity* | 57 (9.9%) | 7 (5.5%) | 50 (11%) | 38 (10%) | 12 (14%) |
| Rheumatoid arthritis/connective tissue disease | 50 (8.7%) | 9 (7.1%) | 41 (9.1%) | 35 (9.6%) | 6 (7.0%) |

^1^Number of comorbidities is the total number of coexisting conditions listed under `Medical history´.

^2^Hospital admissions of more than 12 hours, from 365 days to 14 days prior to the index date.

^3^Current drug use is defined as at least one filled prescription within 6 months prior to the test date.

^4^Medical history is based on an ever-recording of hospital discharge diagnoses. Comorbidities marked by * are defined by hospital discharge diagnoses in combination with drug use for the comorbidity (i.e. filled prescription within 6 months prior to the test date). For details on definitions, see **Supplementary Table S1**.

**These patients died without having a recorded hospital admission (defined as hospitalizations lasting 12 hours or more) within 14 days of the index date. Of note, 17 of these patients were recorded as being admitted to hospital at time of death (mainly due to death occurring very shortly after admission).

***Age categories (0-29, 30-69), and 3 and 4+ hospital admissions within the last year collapsed to ensure anonymity.

****To ensure anonymity, Danish law prohibits reporting of exact n= measures (–) in some cases where this could lead to inferring of low n results (n<5) in other categories.

IQR: interquartile range; SARS-CoV-2: Severe acute respiratory syndrome coronavirus 2. ICU: intensive care unit; ACE: angiotensin-converting enzyme inhibitor; ARB: angiotensin receptor blocker; NSAID: non-steroidal anti-inflammatory drugs.

**Supplementary Table S3.** Predictors of all-cause mortality among hospitalized SARS-CoV-2 PCR-positive cases.

|  | **Death within 30 days^5^** | | |
| --- | --- | --- | --- |
| Characteristics | Crude OR (95%CI) | Age- and sex-adjusted OR (95%CI) | Age-, sex-, and number of comorbidities adjusted OR (95%CI)^1^ |
| **Age, years^1^** |  |  |  |
| 0-9 | NA | NA | NA |
| 10-19 | NA | NA | NA |
| 20-29 | NA | NA | NA |
| 30-39 | NA | NA | NA |
| 40-49 | NA | NA | NA |
| 50-59 | 1.00 (ref.) | 1.00 (ref.) | 1.00 (ref.) |
| 60-69 | 3.5 (1.9-6.4) | 3.4 (1.8-6.3) | 2.9 (1.6-5.4) |
| 70-79 | 7.2 (4.1-12.8) | 7.3 (4.1-12.9) | 5.2 (2.9-9.2) |
| 80-89 | 13.3 (7.5-23.4) | 14.5 (8.2-25.6) | 10.2 (5.7-18.2) |
| 90+ | 32.2 (17.0-61.1) | 39.9 (20.8-76.5) | 29.1 (15.0-56.5) |
| **Sex^1^** |  |  |  |
| Female | 1.00 (ref.) | 1.00 (ref.) | 1.00 (ref.) |
| Male | 1.5 (1.2-1.8) | 1.9 (1.5-2.4) | 1.9 (1.5-2.5) |
| **Authorized health care workers** |  |  |  |
| Non-health care worker | 1.00 (ref.) | 1.00 (ref.) | 1.00 (ref.) |
| Nurse | NA | NA | NA |
| Physician | 0.3 (0.1-1.3) | 1.3 (0.3-6.0) | 1.6 (0.3-7.4) |
| Other | 0.2 (0.0-0.6) | 1.1 (0.3-5.0) | 1.2 (0.3-5.3) |
| **Number of comorbidities^2^** |  |  |  |
| 0 | 1.00 (ref.) | 1.00 (ref.) |  |
| 1 | 3.7 (2.3-6.2) | 2.2 (1.3-3.8) |  |
| 2 | 5.8 (3.5-9.5) | 2.1 (1.2-3.5) |  |
| 3 | 8.7 (5.3-14.2) | 2.9 (1.7-5.0) |  |
| 4+ | 12.8 (7.9-20.5) | 4.1 (2.4-6.8) |  |
| **Hospital contacts within the last year^3^** |  |  |  |
| 0 | 1.00 (ref.) | 1.00 (ref.) | 1.00 (ref.) |
| 1 | 2.4 (1.8-3.1) | 1.6 (1.2-2.1) | 1.4 (1.0-1.8) |
| 2 | 3.3 (2.3-4.7) | 2.3 (1.6-3.4) | 2.0 (1.3-3.0) |
| 3 | 3.5 (2.2-5.5) | 2.3 (1.4-3.8) | 1.9 (1.1-3.1) |
| 4+ | 2.8 (1.9-4.2) | 2.3 (1.5-3.5) | 1.7 (1.1-2.7) |
| **Medical history^4^** |  |  |  |
| Chronic lung diseases* | 1.6 (1.2-2.0) | 1.4 (1.1-1.8) |  |
| Hypertension* | 2.8 (2.2-3.5) | 1.2 (0.9-1.5) |  |
| Ischemic heart disease* | 1.8 (1.4-2.3) | 1.0 (0.8-1.3) |  |
| Heart failure | 2.7 (2.0-3.6) | 1.4 (1.0-1.9) |  |
| Loop-diuretic use** | 3.4 (2.7-4.3) | 1.8 (1.4-2.3) |  |
| Atrial fibrillation | 3.0 (2.3-3.8) | 1.4 (1.1-1.9) |  |
| Stroke | 2.4 (1.8-3.1) | 1.4 (1.0-1.8) |  |
| Diabetes* | 1.7 (1.3-2.1) | 1.4 (1.1-1.8) |  |
| Non-insulin glucose lowering drug use** | 1.5 (1.1-2.0) | 1.3 (1.0-1.8) |  |
| Any insulin use** | 1.9 (1.3-2.7) | 1.5 (1.1-2.3) |  |
| Insulin monotherapy use** | 1.3 (0.7-2.3) | 1.0 (0.5-1.9) |  |
| Dementia* | 5.4 (3.8-7.7) | 2.8 (1.9-4.1) |  |
| Any cancer | 1.9 (1.5-2.4) | 1.2 (0.9-1.6) |  |
| Chronic liver disease | 1.0 (0.5-1.9) | 1.9 (0.9-3.7) |  |
| Hospital-diagnosed kidney disease | 2.6 (1.9-3.5) | 1.7 (1.2-2.3) |  |
| Alcohol abuse* | 1.5 (1.0-2.3) | 1.8 (1.1-2.9) |  |
| Substance abuse* | 1.7 (1.0-3.1) | 1.7 (0.9-3.2) |  |
| Major psychiatric disorder* | 2.3 (1.1-4.8) | 2.9 (1.3-6.6) |  |
| Benzodiazepines and derivates use** | 2.1 (1.6-2.8) | 1.7 (1.3-2.4) |  |
| Antipsychotic use** | 2.8 (1.9-4.1) | 3.3 (2.1-5.1) |  |
| Antidepressant use** | 1.8 (1.4-2.3) | 1.5 (1.1-2.0) |  |
| Organ transplantation | 1.8 (0.7-4.3) | 4.2 (1.6-11.4) |  |
| Medical overweight and obesity* | 0.9 (0.6-1.2) | 1.2 (0.8-1.7) |  |
| Rheumatoid arthritis/connective tissue disease | 1.6 (1.1-2.4) | 1.2 (0.8-1.9) |  |

^1^Age was adjusted for sex and number of comorbidities while sex was adjusted for age and number of comorbidities.

^2^Number of comorbidities is the total number of coexisting conditions listed under `Medical history´.

^3^Hospital admissions of more than 12 hours, from 365 days to 14 days prior to the index date.

^4^Medical history is based on an ever-recording of hospital discharge diagnoses. Comorbidities marked by * are defined by hospital discharge diagnoses in combination with drug use for the comorbidity (i.e. filled prescription within 6 months prior to the test date). ** denotes exclusive use of drugs that are close markers of specific underlying comorbidities, assessed independently of presence or absence of hospital diagnoses for the comorbidity. For details on definitions, see **Supplementary Table S1**.

^5^ Death was defined as all-cause mortality within 30 days from the index date.

OR: odds ratio; SARS-CoV-2: Severe acute respiratory syndrome coronavirus 2.

NA: Not applicable due to too few cases.

Grey boxes for Medical history indicate that ORs for single comorbidities were not adjusted for total number of comorbidities in the main analysis, because some comorbidities may be an effect of the index comorbidity.

**Supplementary Table S4.** Predictors of hospitalization and having a fatal course within 30 days among SARS-CoV-2 PCR-positive cases, when adjusting single comorbidities for age-, sex-, and additionally for total number of comorbidities.

|  | **Hospitalization** | **Death within 30 days^2^** |
| --- | --- | --- |
| Characteristic | Age-, sex., and number of comorbidities adjusted OR (95%CI) | Age-, sex., and number of comorbidities adjusted OR (95%CI) |
| **Medical history^1^** |  |  |
| Chronic lung diseases* | 1.2 (1.1-1.5) | 1.1 (0.8-1.4) |
| Hypertension* | 0.9 (0.8-1.1) | 0.6 (0.5-0.8) |
| Ischemic heart disease* | 0.9 (0.7-1.0) | 0.7 (0.5-0.9) |
| Heart failure | 1.6 (1.2-2.2) | 1.1 (0.8-1.5) |
| Loop-diuretics | 1.8 (1.5-2.3) | 1.7 (1.3-2.1) |
| Atrial fibrillation | 0.9 (0.7-1.1) | 1.1 (0.8-1.4) |
| Stroke | 0.9 (0.7-1.1) | 1.0 (0.8-1.3) |
| Diabetes* | 1.1 (0.9-1.4) | 1.1 (0.8-1.4) |
| Non-insulin glucose lowering drugs | 1.1 (0.9-1.3) | 1.0 (0.7-1.3) |
| Insulin | 1.4 (1.0-1.8) | 1.3 (0.9-1.8) |
| Insulin monotherapy | 1.4 (0.9-2.2) | 1.0 (0.6-1.7) |
| Dementia* | 0.4 (0.3-0.6) | 1.7 (1.3-2.2) |
| Any Cancer | 1.0 (0.8-1.2) | 1.0 (0.8-1.3) |
| Chronic liver disease | 1.4 (0.9-2.0) | 1.2 (0.7-2.3) |
| Hospital-diagnosed kidney disease | 1.8 (1.3-2.4) | 1.3 (0.9-1.8) |
| Alcohol abuse* | 1.1 (0.8-1.4) | 1.3 (0.9-2.0) |
| Substance abuse* | 0.8 (0.5-1.1) | 1.3 (0.7-2.2) |
| Major psychiatric disorder* | 1.4 (0.8-2.4) | 1.9 (0.9-3.9) |
| Benzodiazepines and derivates | 1.5 (1.2-1.8) | 1.8 (1.4-2.4) |
| Antipsychotics | 1.2 (0.9-1.6) | 3.0 (2.1-4.2) |
| Antidepressants | 1.1 (0.9-1.3) | 1.5 (1.2-1.9) |
| Organ transplantation | 1.7 (0.9-3.3) | 2.0 (0.8-5.1) |
| Medical overweight and obesity* | 1.3 (1.1-1.6) | 1.0 (0.7-1.4) |
| Rheumatoid arthritis/connective tissue disease | 1.0 (0.8-1.3) | 0.9 (0.6-1.3) |

^1^Medical history is based on an ever-recording of hospital discharge diagnoses. Comorbidities marked by * are defined by hospital discharge diagnoses in combination with drug redemptions (i.e. filled prescription within 6 months prior to the test date. For details on definitions, see **Supplementary Table S1**.

^2^Death was defined as all-cause mortality within 30 days from the index date.

OR: odds ratio; SARS-CoV-2: Severe acute respiratory syndrome coronavirus 2.

**Supplementary Table S5.** Clinical characteristics of SARS-CoV-2 PCR-positive cases during subperiods of the epidemic, i.e. containment phase, mitigation phase, and during reopening of the society.

|  | Containment phase^1^  27^th^ Feb to 11^th^ Mar | Mitigation phase^2^  12^th^ Mar to 14^th^ Apr | During gradual re-opening^3^  15^th^ Apr onwards |
| --- | --- | --- | --- |
| All individuals tested (n) | 3326 | 77672 | 340821 |
| PCR-positive cases (n) | 756 | 6,059 | 4,307 |
| Mortality (%) | 0.8% | 6.7% | 3.8% |
| Age, median [IQR] | 43 (32-50) | 52 (38-66) | 44 (28-60) |
| ≤18 years | 30 (4.0%) | 183 (3.0%) | 446 (10%) |
| 19-59 years | 671 (89%) | 3,759 (62%) | 2,773 (64%) |
| 60-74 years | 49 (6.5%) | 1,155 (19%) | 584 (14%) |
| ≥75 years | 6 (0.8%) | 962 (16%) | 504 (12%) |
| Sex |  |  |  |
| Female | 243 (32%) | 3,598 (59%) | 2,589 (60%) |
| Male | 513 (68%) | 2,461 (41%) | 1,718 (40%) |
| Health care workers (N) | 50 (6.6%) | 1,483 (24%) | 894 (21%) |
| Number of comorbidities | 0 (0-1) | 1 (0-2) | 0 (0-1) |
| 0 | 557 (74%) | 2,987 (49%) | 2,490 (58%) |
| 1 | 157 (21%) | 1,388 (23%) | 917 (21%) |
| 2 | 33 (4.4%) | 709 (12%) | 398 (9.2%) |
| 3 | 9 (1.2%)* | 460 (7.6%) | 225 (5.2%) |
| 4+ |  | 515 (8.5%) | 277 (6.4%) |

^1^Test strategy during the containment phase: mainly testing of suspected symptomatic COVID-19 cases with a relevant travel history (mainly from China and Italy)

^2^Test strategy during the mitigation phase: Initially testing of individuals with suspected COVID-19 requiring hospital admission, and testing of symptomatic frontline health care workers in critical functions (e.g. ICU personnel). From late-March onwards, upscaled to include testing of individuals with mild to moderate respiratory symptoms suspicious of COVID-19, as well as broader screening of healthcare professionals

^3^Test strategy during re-opening: Gradually upscaled to include testing of any individuals with mild to severe symptoms suspicious of COVID-19.

*Number of comorbidities (3 and 4+) collapsed to ensure anonymity.

SARS-CoV-2: Severe acute respiratory syndrome coronavirus 2.

**Supplementary Figure S1.** Hospitalization (A), ICU admission (B) and death in timely relation to the date of having a positive test for SARS-CoV-2 (C).

1. SARS-CoV-2 PCR-positive cases who were hospitalized: date of hospital admission relative to sample date


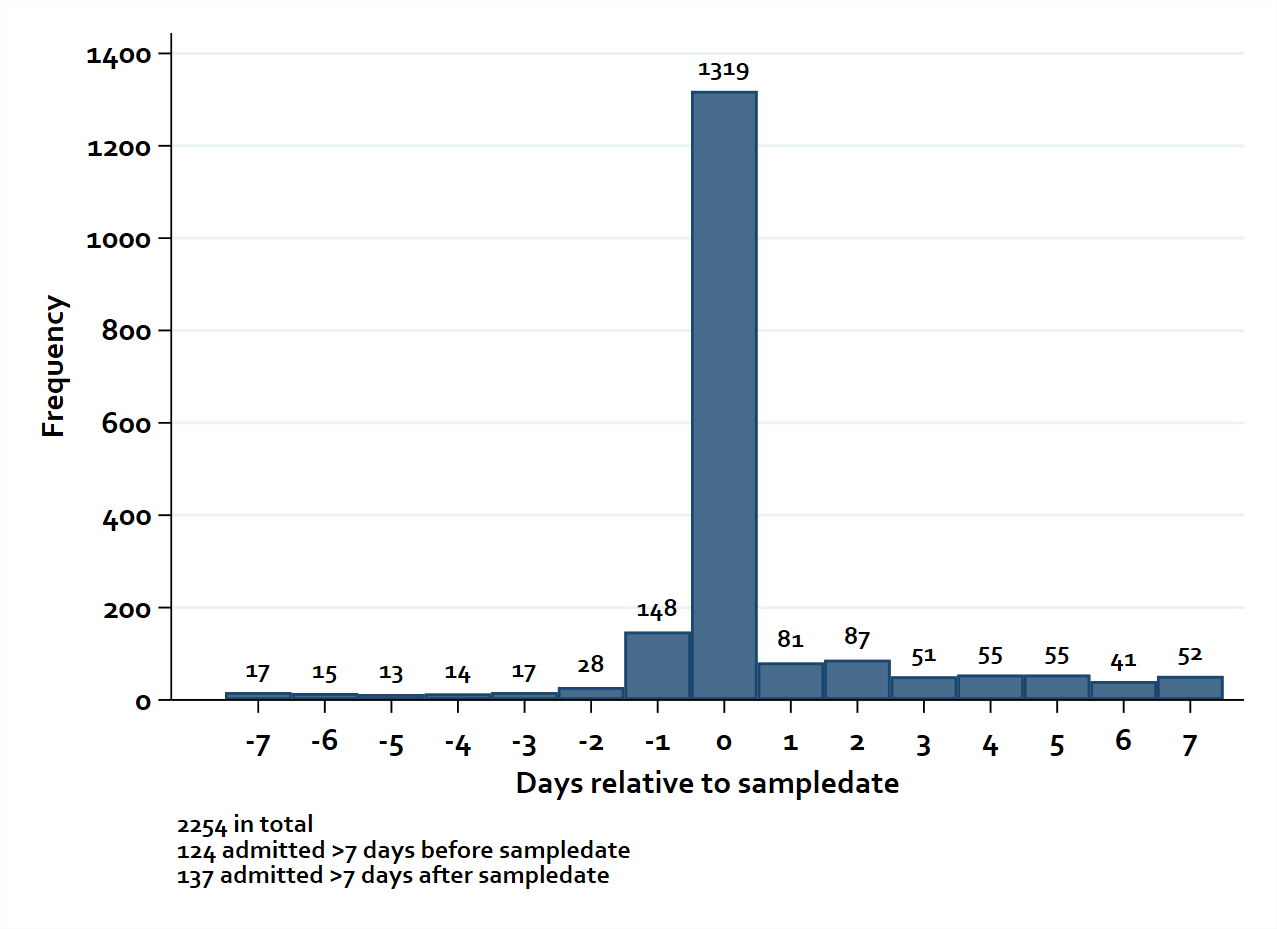


B) SARS-CoV-2 PCR-positive cases who were admitted to ICU: date of ICU admission relative to sample date


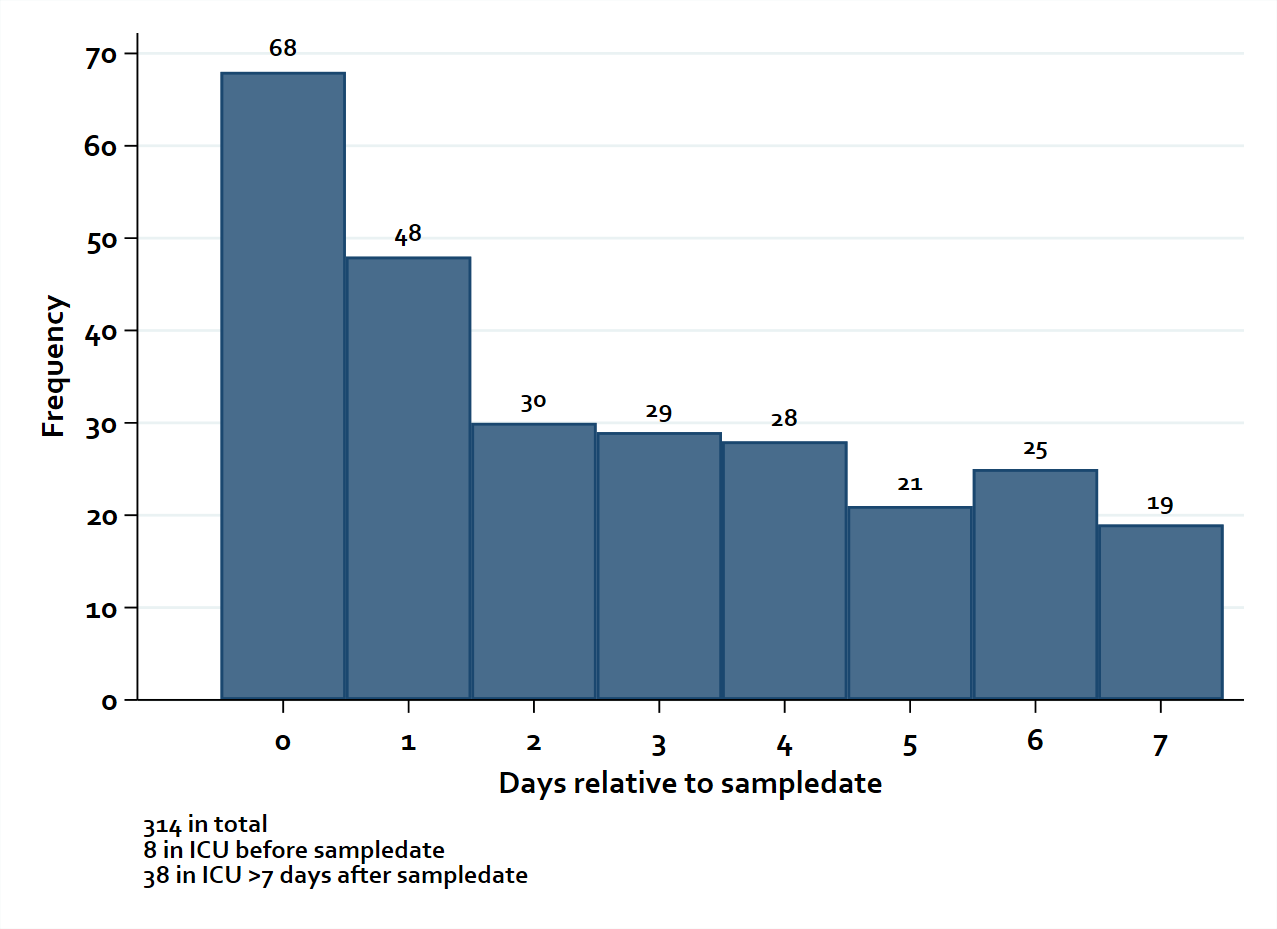


C) SARS-CoV-2 PCR-positive cases who died: date of death relative to sample date


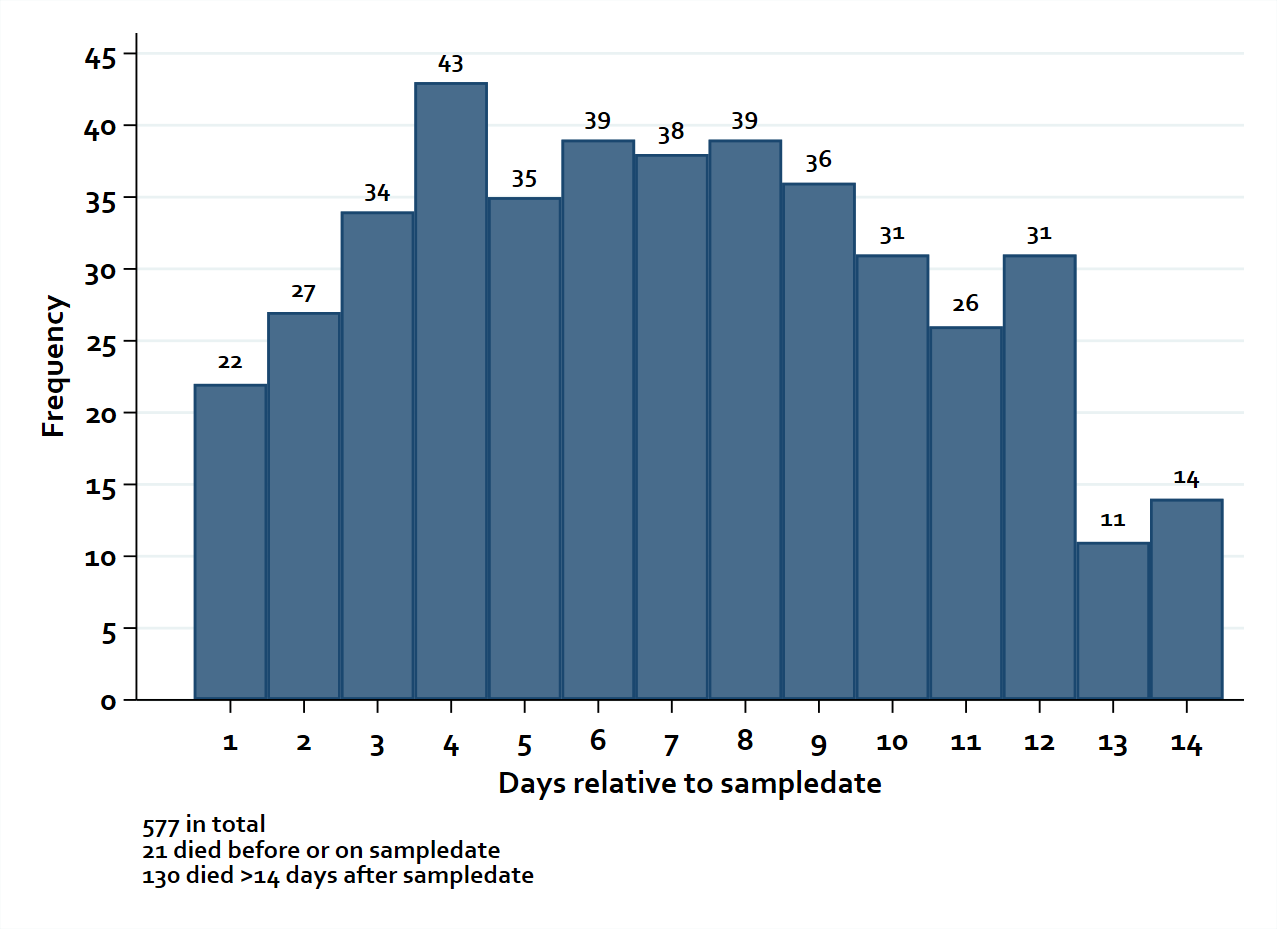

Supplement: dyaa140_supplementary_data [file dyaa140_supplementary_data.docx]
